# Socioeconomic Drivers of Adoption and Use Intensity of Improved Teff Seed in Northwest Ethiopia

**DOI:** 10.1002/fsn3.71030

**Published:** 2025-09-26

**Authors:** Gizachew Wosene, Yuhan Pang, Jianmin Cao, Mezgebu Aynalem, Arshad Ullah Jadoon

**Affiliations:** ^1^ School of Economics and Management Jilin Agricultural University Changchun China; ^2^ Department of Agribusiness and Value Chain Management Debre Markos University Bure Campus Burie Ethiopia

**Keywords:** adoption, double‐hurdle model, Ethiopia, teff

## Abstract

This study investigates the key factors influencing both the decision to adopt and the level of use of improved teff seed technology among farmers in the Jabitehnan district, Northwest Ethiopia. Despite teff being a vital staple cereal crop extensively cultivated in Ethiopia, it shows inconsistent yields mainly due to a combination of demographic, socioeconomic, and institutional factors. A two‐stage random sampling method was used to gather data from a sample of 384 respondents, of whom 276 (71.87%) were adopters and 108 (28.13%) were nonadopters. Descriptive and inferential statistics, including *t*‐tests and chi‐square tests, were employed to characterize the selected households. Furthermore, Cragg's double‐hurdle econometric model was utilized to analyze the determinants affecting household adoption and the intensity of improved teff seed use. The results from the first hurdle (adoption decision) indicate that farming experience, education level, farm size, participation in demonstration plots, attendance at field days, family size, frequency of extension contacts, and household income all have positive and significant effects on the likelihood of adoption. Conversely, the age of the household has a negative effect. The second hurdle (intensity of adoption) results suggest that the level of improved teff seed use is similarly positively influenced by education, farm size, participation in demonstrations and field days, extension contacts, and income, while age remains negatively associated. Based on these findings, researchers, policymakers, extension service providers, and development organizations focused on agriculture should prioritize these key factors to promote the adoption and intensive use of improved teff technologies.

## Graphical abstract

Data from 384 households (276 adopters, 108 nonadopters) was analyzed using descriptive statistics and Cragg's double‐hurdle model. Results show that adoption is positively influenced by farming experience, education, farm size, demonstration participation, field day attendance, family size, extension contacts, and income, whilst age has a negative impact. Similarly, the use intensity of improved teff seed was driven by education, farm size, demonstrations, field days, extension contacts, and income, with age again exerting a negative influence. Policy recommendation: Based on these findings, researchers, policymakers, extension service providers, and development organizations focused on agriculture should prioritize these key factors to promote the adoption and intensive use of improved teff technologies.

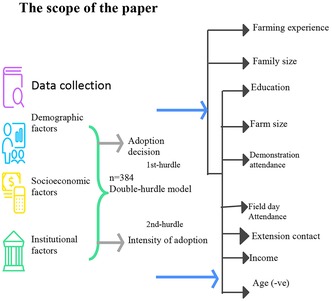

**Abbreviations**
BoA: Bureau of Agriculture
CDF: cumulative distribution function
CSA: Central Statistical Agency
FGD: focus group discussion
GDP: Gross Domestic Product
SARC: Sirinka Agricultural Research Center

## 1 Introduction

### 1.1 Background and Justification

Agriculture sector in Ethiopia plays a key role in providing the livelihood of 80% of the population. It accounts for 34.1% of the GDP, 79% of employment, and 79% of foreign earnings. Despite its importance, the sector is dominantly characterized by low productivity, subsistence production, and a traditional farming system (Zegeye 2021).

Enhancing agricultural productivity, ensuring food security, and alleviating poverty heavily depend on the use of improved agricultural technologies. These technologies include high‐yield seed varieties, irrigation systems, pesticides, fertilizers, plowing equipment, harvesting and threshing machines, as well as modern agronomic practices (Assefa and Gezahegn 2011). Although the Ethiopian government has invested considerable effort in promoting and expanding access to these technologies, the actual adoption rate among farmers remains relatively low. This limited uptake continues to hinder potential gains in agricultural output (Natnael 2019).

Among the various improved agricultural technologies initiated and promoted in Ethiopia, improved *teff* seed varieties stand out as a pivotal point for addressing productivity and food security issues. To this end, this study examines the factors influencing the adoption and intensity of adoption of improved *teff* seed varieties in northwest Ethiopia. *Teff* (*Eragrostis teff*), *a nutrient‐rich cereal native to* Ethiopia, is a staple food and major economic contributor, making up 6.1% of real GDP (Fantu et al. 2015). In the 2020–2021 production season, cereals cover 81.19% (10,538,341.91 ha) of the total cereal crop area. *Teff*, maize, sorghum, and wheat comprised 22.56% (approximately 2,928,206.26 ha), 19.46% (approximately 2,526,212.36 ha), 12.94% (1,679,277.06 ha), and 14.62% (1,897,405.05 ha) of cereal crop area, respectively (CSA 2020).

Yet, despite the *teff* crop is broadly cultivated and has significant importance in Ethiopia, the traditional production techniques and limited access to improved inputs have limited its ability to meet the growing consumer demand (Tesfay and Gebresamuel 2016). The persistence of these constraints highlights the urgent need for improved production techniques and technologies that are tailored to the local context. In this regard, context‐specific innovations—such as the improved *teff* seed packages introduced in Ethiopia—have the potential to significantly enhance adoption among smallholder farmers. When agricultural interventions are aligned with the unique conditions and needs of farming communities, they tend to be more effective and sustainable. Supporting evidence from Kenya also illustrates how regionally adapted agricultural practices can drive technology uptake, as shown by (Kimaru‐Muchai et al. 2021). These insights underscore the critical role of localized training, input systems, and extension services in promoting the adoption of improved *teff* varieties and, ultimately, in addressing the broader challenges of low productivity and food insecurity.

To enhance *teff* yield, Ethiopia has introduced several improved *teff* seeds for a long time with their better management practices package (SARC 2015). However, various area‐specific studies indicate that improved *teff* seed adoption rates remain low. Farmers' adoption decisions are often influenced by multiple factors. Limited access and high cost of improved seed, limited know‐how, and stringent agronomic requirements also count as limiting factors for the adoption of improved *teff* seeds (Berihun et al. 2014).

In light of these barriers, it is important to investigate how specific socioeconomic and institutional factors determine and shape the adoption behaviors of smallholder farmers. One way to boost farm‐level productivity is by introducing and distributing enhanced farming inputs to rural households (Workie and Tasew 2023). They found that farmers with greater access to the extension system, larger land holdings, and proximity to markets are willing to use the newly released technology package. Other related studies have also identified influencing factors for the utilization of enhanced agricultural input by rural households (Teshome and Tegegne 2020) and factors influencing enhanced *teff* seed adoption and its effect on output per input (BoA 2019). The study by (Tekeste et al. 2023) has examined the factors influencing the adoption of certified maize, *teff*, and wheat using a logit model. Their findings indicate that variables such as education, farm size, experience, income, credit access, extension context, farm input, and distance to the market significantly influence the adoption decision of improved seeds. However, their study does not address the extent or intensity of adoption.

Similarly, other related studies by (Ayal et al. 2018; Fikire and Asefa 2023) focused on determinants of adoption of *teff* row planting in Ethiopia using a logit model, but do not examine the adoption and extent of use of improved *teff* seeds. The study by (Beshir et al. 2012) investigates the determinants of land allocation decisions and the intensity of land allocation for Tef‐*Acacia decurrens‐charcoal* production agroforestry using a double hurdle model. However, it did not examine the adoption and intensity of adoption of improved *teff* seeds. (Nonvide 2021) investigates determinants of adoption of agricultural technologies among rice farmers in Benin using a probit model which is methodologically, spatially, and institutionally different from the current studies. Therefore, a comprehensive approach to address these gaps is needed. Specifically, there are limited studies that simultaneously analyze both the decision to adopt and the extent of adoption of improved *teff* seeds.

Therefore, the present study generally aims to examine socioeconomic factors influencing the adoption of improved agricultural technologies. Specifically, it aims to investigate spatial‐specific factors influencing both the adoption and intensity of use of improved *teff* seeds such as Quncho, Tsedey, and Dega in northwest Ethiopia. To achieve this, the study employs a double‐hurdle model, which is well suited to capture these two distinct, related aspects of adoption behavior. Ultimately, the study aims to generate valuable, area‐specific knowledge that can support augmented *teff* production, identify and address existing challenges, and leverage untapped opportunities. Moreover, the findings of this research will provide significant remarks for policy implications and development practitioners, contributing to the design of more effective and sustainable agricultural strategies that enhance *teff* productivity across diverse regions of the country.

## 2 Literature Review

### 2.1 Concept and Definition of Adoption

According to (Feder et al. 1985), adoption is a mind activity an individual goes through learning about an innovation to putting it into practice. This term is considered to classify adopters as those who have used and are still using technology, while nonadopters are individuals who have never used technology or who have just used it once. Adoption and diffusion are phrases that are used interchangeably, according to data from the literature. According to (Rogers 2003), adoption is the mental process through which an individual passes from first hearing about an innovation to final adoption. The adoption process comprises five stages, including awareness, interest, evaluation, trial, and adoption, in relation to this concept.

The other authors defined the adoption of improved agricultural technology as a farm household that has been using one or more of a given improved agricultural technology consistently for at least 2 years (Wordofa et al. 2021). Therefore, if a producer farmer is found to be growing any improved *teff* seeds (ITS), they are considered adopters. When studying adoption, some thought was given to how to define which technologies are to be considered.

### 2.2 Theoretical Framework of Improved *Teff* Seed Adoption

Although there are a number of theories and models of adoption such as the Theory of Diffusion of Innovation (TDI), the Technology Acceptance Model (TAM), the Unified Theory of Acceptance and Use of Technology (UTAUT), Technology Readiness (TR), the Theory of Planned Behavior (TPB), the Digital Business Model (Göcke and Weninger 2021; Prasetyani et al. 2025), and the Utility Maximization Theory (Dissanayake et al. 2022; Lai 2017), this study adopted the Random Utility Model due to its practical application to the farmer situation (Atube et al. 2021; Midamba et al. 2024).

Random Utility Model Theory indicates that the farmers' adoption decision is based on the perceived benefits of any given technology (Kanyamuka 2017; Lai 2017). Farmers obtain information on the existence of a technology before implementing any ITS. The farmer then evaluates the technology against the existing ones while considering other factors such as the costs of the technology, trial ability and relative advantage (Scott et al. 2008). Consequently, a farmer would then adopt any given technology if they perceive such a technology as beneficial. A rational smallholder farmer, seeking to increase *teff* production, considers adopting an improved teff seed varieties. Farmers adopt improved *teff* seeds (ITS) which give the highest utility (chooses *ITS* if *Ui*_*MT*_>*U*_*ik*_) (Ngoma et al. 2016). Random Utility Model (RUM) is specified in the equation below:

(1) $$U_{j}=\text{βjXi}+\mathrm{\varepsilon i}$$

In the above equation, *Uj* is the perceived utility of a given agricultural technology; it is it also hypothesized that the adoption decision is influenced by socioeconomic factors which are represented by *Xi*, while *βj* are parameters to be estimated, and *εi* represents the error term.

### 2.3 Empirical Studies

Empirically, different research works have been conducted to investigate the determinants of adoption and intensity of use of improved agricultural technologies in Ethiopia.

(Zenbaba et al. 2024) examine the factors influencing the adoption of wheat production technology packages by smallholder farmers in Ethiopia using a two‐limit Tobit model. The results indicate that factors such as the household head's education level, access to credit, access and purchase of improved seed, livestock owned, farmer's perception of wheat yield, farm training received, and annual farm income are positively and significantly associated with the adoption intensity of wheat technology packages. In contrast, distance from the nearest market had a negative and significant effect. (Ibrahim et al. 2024) investigate the key factors influencing the adoption of improved wheat production technologies in Sudan's irrigated, heat‐prone, arid environments. The binary logistic regression results revealed that access to quality seeds, financial credit, and extension services were found to be the most critical determinants of adopting improved technologies.

(Guye et al. 2024) used a multivariate probit model to analyze the determinants of multiple maize technology package adoption in Ethiopia. The study revealed that farming experience, family size, plot size, livestock and oxen ownership, the number of maize plots owned, off‐farm income, access to credit, extension services, and membership in institutions are determinant factors affecting the likely to adopt at least one of the improved technology packages and achieved a better status of intensity of adoption. The study from Côte d'Ivoire examined the mobile money users and factors influencing the adoption process of mobile money technology. The results from the logit model indicate that demographic factors such as age, sex, and marital status; social factors such as membership in agricultural cooperatives, belonging to saving and credit groups; and institutional factors including the level of education and being customers of banks or microfinances (Nonvide and Alinsato 2023).

According to (Siyum et al. 2022), the double‐hurdle model was applied to investigate the probability and intensity of use of improved bread wheat varieties and associated technologies. The results revealed that oxen, phone ownership, education level, and extension service influence the likelihood of adoption, while land tenure, demonstration participation, awareness of the shattering problem of local bread wheat varieties, and household income statistically and significantly influence the intensity of adoption of improved bread wheat varieties. The study by (Zegeye et al. 2022) employed a multinomial logit model to evaluate the determinants influencing farmer decision to adopt multiple agricultural technologies. The findings indicate that farmers with more educational level, family size, off‐farm participation, livestock, extension service, credit access, advisory service, and farmers closer to plot, all‐weather road, zonal town, and farmers with lower remittance income are more likely to adopt new or improved agricultural technology.

(Kassa et al. 2021) examine determinants of adoption and intensity of improved Faba bean cultivars in the central highlands of Ethiopia using a double‐hurdle model. The results show that family size, farmer awareness of improved cultivars, and extension contact significantly influence the adoption decision of improved Faba bean cultivars, while livestock holding and access to market information affect the intensity of adoption. The study by (Zeleke et al. 2021) used a double‐hurdle model to examine the effect of key variables on the adoption of improved wheat varieties. The estimated results revealed that farming experience, distance to cooperative, renting a tractor and combiner harvest, urea, and net income significantly influence the likelihood of the adoption decision of improved wheat varieties.

(Kebede et al. 2017) investigate the factors influencing the adoption of the wheat production technology package among smallholder farmers in eastern Ethiopia. The findings of the two‐limit Tobit model revealed that several factors significantly impacted adoption, including district variation, gender, age of the household head, education status of the household head, farm size, distance to market, distance to FTC (Farmers' Training Centers), cooperative membership, dependency ratio, and annual income of the households, which were found to significantly affect the adoption of wheat technology packages.

### 2.4 Conceptual Framework

**FIGURE 1:**
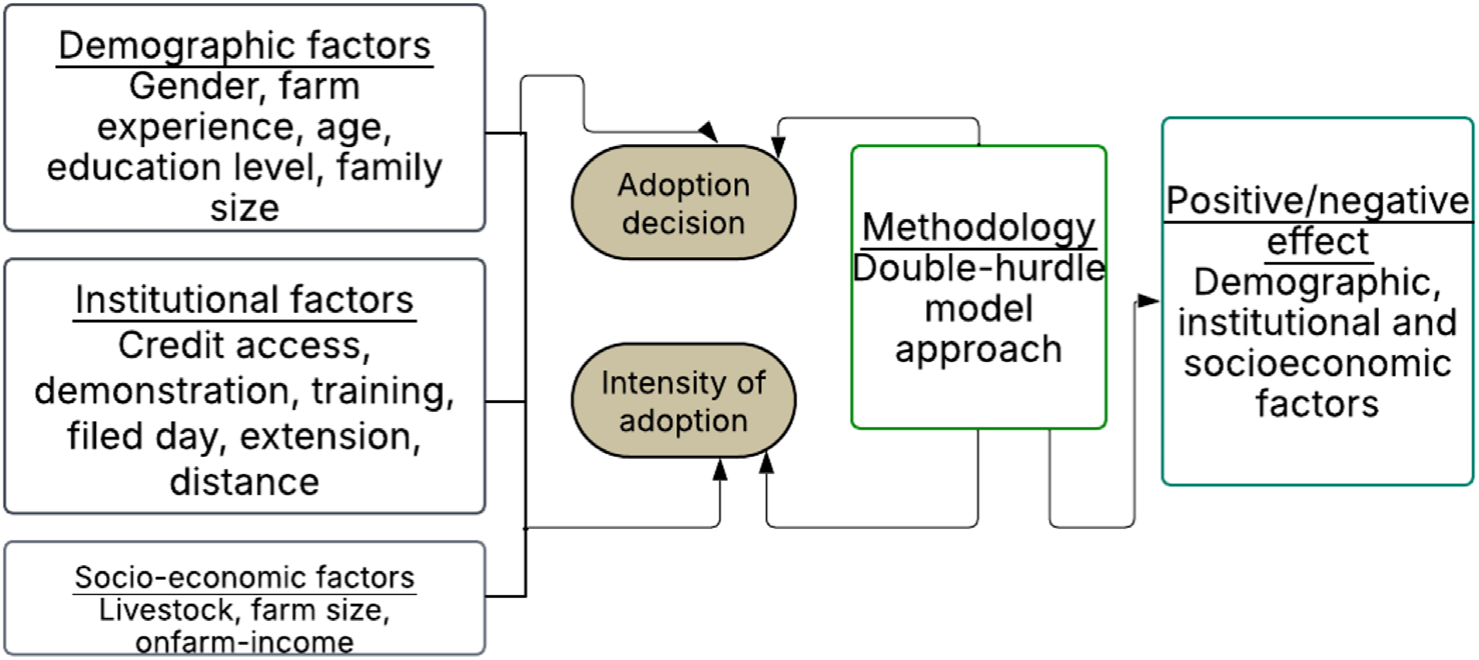
Conceptual framework of the study.

We used Rogers'diffusion theory as a reference to develop this study's conceptual framework, which considers technology adoption as a process influenced by multiple interconnected factors. The framework categorized these interconnected factors into three major groups, including demographic, socioeconomic, and institutional factors in the context of improved *teff* seed adoption among smallholder farmers in Northwest Ethiopia. Demographic factors such as age, education level, family size, and farming experience are expected to influence both the decision to adopt and the intensity of use, as they affect farmers' opinions on innovation and their ability to apply new practices (Emerick and Dar 2021). Socioeconomic factors, including farm size, livestock ownership, on/off‐farm income, and resource capacity of farmers, influence farmers to invest in agricultural innovations (Mgendi et al. 2022). Institutional factors, including extension contact, participation in demonstration and field days, access to credit, and distance from the seed sources, also play a key role in shaping adoption decisions and the intensity of use of agricultural technologies (Workineh et al. 2020). A two‐step adoption process model framework is assumed through a double hurdle approach: first, a binary choice of farmer adoption decision, and second, intensity of adoption measured by the amount of seed used per hectare. The interaction of these variables determines both whether a household adopts and how extensively the technology is used (Figure 1). This framework serves as a basis for empirical analysis and policy recommendations, recognizing that adoption is not only a function of access to inputs but also of the broader demographic and institutional environment in which farmers operate.

## 3 Research Methods

### 3.1 Area Description

Jabitehinan district is one of the 15 districts in the West Gojjam Zone, located 374 km away from the southwest of Bahir Dar, the regional capital. The Woreda covers a total area of 117,020 ha. Currently, it is divided into 38 rural kebele administrations and four towns. Finoteselam, Jiga, and Mankusa are the major towns in the Woreda. According to the 2019 report from the Woreda Bureau of Agriculture, the total population of the Woreda is 270,147, with 253,348 residing in the countryside and the remaining 16,799 in urban areas (BoA 2019).

The climate of Woreda is generally 88% *Weyna Dega* and 12% *Kola*. The Woreda receives an average of 1250 mm of rainfall per year. It is located at an altitude of 10° 42′ N and a longitude of 37° 16′ E with an elevation of 1917 m in elevation. The yearly average rainfall is 1450.3 mm, with the mean minimum and maximum temperatures being 10°C and 23°C, respectively. The soil type is nitosol, with a pH ranging from 5.3 to 6.

### 3.2 Types and Sources of Data

The primary source of data was employed for the investigation. *Teff* producer rural households, agricultural experts, and kebele leaders were considered to gather primary sources of information. Quantitative data were obtained using a structured questionnaire administered to *teff* producers, while qualitative data were collected from focus group discussions (FGD) via interviewing. Potential participants for the FGD were selected from among the chosen kebeles.

### 3.3 Sampling Techniques and Sample Size

**FIGURE 2:**
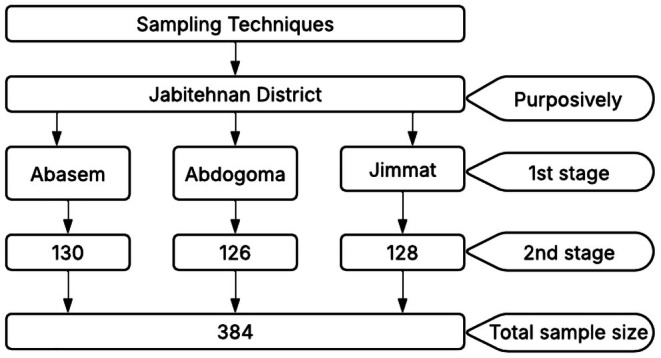
Sample size determination procedure.

Jabitehnan was selected purposefully due to its potential for *teff* production in West Gojjam Zone. A two‐stage random sampling technique was adopted to select the sampled *teff* producers. In the first stage, in Jabitehnan district, three *teff* producers' kebeles, namely Abasem, Jimmat, and Abdogoma, were randomly selected. In the second stage, from these three kebeles, 384 sampled rural households were randomly selected based on proportional sampling to size (Figure 2).

To determine the representative sample size from the large population, the Cochran (1977) formula was applied. Hence, the sample size is estimated as:

$$n=\frac{Z^{2}.P.\left(1-P\right)}{{\left(e\right)}^{2}}=\frac{1.96^{2}.0.5.\left(1-0.5\right)}{{\left(0.05\right)}^{2}}=384$$

*n*—Required sample size.

*Z* = *Z*‐value (from the standard normal distribution at 95% CI, 1.96).

*P* = estimated proportion of the population (use 0.5 if unknown, as it gives the maximum variability).

*e* = desired level of precision (accepted margin of error, 5%).

### 3.4 Methods of Data Collection

A total of 384 households across three sample kebeles were selected for the study. To understand the socioeconomic determinants of adoption and intensity of use of improved *teff* seed, this study relied on primary data sources. Structured household surveys using pre‐tested questionnaires were the primary tool, allowing for the collection of quantitative data on demographics, socioeconomic, and institutional factors. Data collection involved face‐to‐face interviews with sampled households, with each session lasting 30–50 min. To complement this, focus group discussions and key informant interviews were also conducted with farmers, extension agents, and local agricultural officials to gain qualitative insights into adoption behavior and contextual factors. Field observations were also carried out to validate the accuracy of the reported practices.

### 3.5 Methods of Data Analysis

In this study, we first used SPSS to organize, enter, and code the survey data in a structured and reliable manner. Once the data were cleaned and prepared, we exported it to Stata version 17 for the statistical analysis. Descriptive statistics were generated to provide an overview of the characteristics of the sample respondents, offering insights into their demographics, socioeconomic, and institutional patterns of the study area. To explore the factors influencing both the decision to adopt improved *teff* seed and the extent of its use, we employed an econometric technique, the double‐hurdle model. This model is particularly well suited for analyzing decisions that involve two separate processes: whether to adopt and how much to use. We implemented this model in Stata using the “crhurdl” command, which allowed us to capture the nuances in farmer behavior and identify key determinants driving adoption.

#### 3.5.1 Descriptive Statistics

To characterize the sample household units, descriptive statistics were employed. Measures such as mean, standard deviation, and percentage were used to describe socioeconomic and institutional characteristics of *teff* producers. In addition, statistical tests like chi‐square and *t*‐test were applied to compare and contrast different categories of sample units concerning the desired character, allowing to make meaningful conclusions.

#### 3.5.2 Econometric Analysis

Various academics have analyzed the factors influencing the adoption of technology using different models. The choice to adopt and the amount to adopt can, in theory, be made either simultaneously or independently (Gebremedhin and Swinton 2003). The Tobit model was used to analyze the two decisions, under the assumption that both were affected by the same set of factors (Greene 2008). The Tobit model, which is an extension of the probit model, can be an alternative solution to address the issue of censored data (Johnston and Dinardo 1997). In contrast, the double‐hurdle model has equations for both hurdles that account for the circumstances and characteristics of the farmer. These explanatory factors could be present in one or both equations (Hailemariam et al. 2006). Additionally, empirical research has shown that a variable that appears in both equations may have different impacts in each equation. Many scholars devised the double‐hurdle model, which has been widely used in a number of empirical studies, including (Cragg 1971), (Hailemariam et al. 2006), (Burton et al. 1996), and (Berhanu and Swinton 2003).

To investigate the effect of the demographic (gender, age, farm experience, education level, and family size), socioeconomic (livestock holding, farm size, on‐farm income), and institutional (credit, participation in demonstration, training, field day, extension contact) factors on both the adoption decision and intensity of adoption of improved *teff* seeds, a double‐hurdle model was employed. The double‐hurdle model, introduced by Cragg (1971), is a two‐step estimation procedure used to analyze decisions on two distinct stages. In the context of agricultural technology adoption, the first stage assesses whether a farmer decides to adopt improved *teff* seeds. In our dataset, the adoption variable is coded as zero for farmers who did not adopt improved *teff* seeds, while it takes on positive continuous values for adopters, representing different levels of adoption intensity. In this stage, a typical model is a probit model, which helps to estimate the probability of adoption based on a set of explanatory variables. The following section presents the standard probit model used to analyze household adoption decisions.

##### 3.5.2.1 1st Stage

The binary outcome variable of adoption decision is denoted by *Y*; then the probit model can be expressed as:

$$Y=\left\{\begin{array}{c} 1\;\text{if farmer adopt improved}\;\mathrm{tef}\;\text{seeds}\\ 0\;\text{if otherwise}\; \end{array}\right.$$

The probit model assumes that the probability of the outcome *Y* = 1 is determined by a latent (unobserved) continuous variable Y*, which is modeled as

$${\mathrm{Yi}}^{*}=\beta x_{i}+e_{i}$$

where Yi* is the latent variable indicating the decision to adopt improved *teff* seeds, *Xi* set of 1 by *k* vector of explanatory variable affecting the decision to adopt an improved *teff* seed of rural household (*i*), *β* is a set of 1 by *K* vector of parameters, and *e*_i_ ~ *N* (0,1) error term, assumed to follow a standard normal distribution.

The observed binary outcome *Y* is related to the latent variable Y* as follows

$$Y_{i}=\left\{\begin{array}{c} 1\;\text{if}\;Y*>0\\ 0\;\text{if}\;Y*\leq 0 \end{array}\right\}$$

Thus, the probability that *Y* = 1 is:

$$\left(\mathrm{Pr}\left(Y=1|x\right)=\mathrm{Pr}\left(Y*>0|x\right)=\mathrm{Pr}\left(\varepsilon >\beta x\right)=\Phi \left(\beta x\right)\right)$$

where *Φ*(·) denotes the cumulative distribution function (CDF) of the standard normal distribution.

##### 3.5.2.2 Second Stage

In the 2nd stage of the double‐hurdle model, a truncated regression model is used to analyze factors determining the intensity of improved *teff* seeds adoption measured in kg per hectare. This stage focused on the sample households that have already decided to adopt the improved *teff* seed (*Yi* = 1) (Wooldridge 2002). The truncated regression model explicitly considered the fact that the sample is limited to adopters, and the dependent variable (intensity of adoption) is truncated at zero:

$${\mathrm{Zi}}^{*}=\beta x_{i}+\mu_{i,}\;\mu_{i}\sim \left(0,\sigma^{2}\right)$$

$$\mathrm{Zi}*=\left\{\begin{array}{c} \mathrm{Zi}*\text{if}\;\mathrm{Zi}*>0\;\text{and}\;\mathrm{YI}=1\\ 0\;\text{otherwise} \end{array}\right\}$$

where the adoption intensity of *teff* seed is denoted as *Z*_i_, a function of the latent variable Z*. The intensity of adoption is observable if the latent variable Z* is greater than zero, and conditional on the rural household having already made the decision to adopt improved *teff* seed (i.e., *Yi* = 1 in the first stage).

The error terms are considered to be independently distributed and normally distributed as follows if each farmer makes both decisions on their own: u𝑖 ~ *N* (0, *σ* ^2^). By Cragg (1971), the log‐likelihood serves as the double‐hurdle model that nests a univariate probit model and a truncated regression model by:

$$\text{LogL}=\sum \mathrm{Ln}\left[1-Ф\left(Z^{\prime }\mathrm{i\alpha}\right)\left(\;\frac{\beta X^{\prime }i}{\sigma }\right)\right]+\sum \ln\;\left[Ф\left(Z^{\prime }\mathrm{i\alpha}\right)\;\frac{1}{\sigma }\phi \;\left(\frac{\mathrm{yi}-{\mathrm{\beta X}}^{\prime }i}{\sigma }\right)\right]$$

Here, *Z*′I and *X′*I represent independent variables for the probit model and the Truncated model, respectively. The parameters *α*, *σ*, and *β* are estimated for each model, while *Ф* and *ϕ* denote the standard normal cumulative distribution function (CDF) and the standard normal density function (PDF), respectively.

### 3.6 Variable Description and Hypothesis

The dependent variable of the probit model has a dichotomous value depending on the farmers' decision either to adopt or not to adopt the improved *teff* seeds. However, the truncated regression model would have a continuous value, which should be the intensity, the amount of *teff* seed used in kg per hectare. Variable description and hypothesis are presented in Table 1.

**TABLE 1:** Definition and hypothesis of working variables for analyses.

Variables	Description and measurement	Expected sign (adoption/intensity)
Decision to adopt (Ado_dic)	Dummy (1 if user, 0 otherwise)	−−−/−−−
Adoption intensity (Ado_level)	Kilogram per hectare (kg/ha)	−−−/−−−
Gender	Dummy (1 male, 0 otherwise)	+/+
Family size (famsz)	Number of family members	+/+
Distance to seed sources (Dist)	Walking hour	−/−
Education (EDU)	Dummy (1 literate, 0 otherwise)	+/+
Age	Age of household head (years)	+/+
Land Holding (LH)	Hectares (ha)	+/+
Farming Experience (Expre)	Year of farming (year)	+/+
Access to credit (AC)	Dummy (1if accessed, 0 otherwise)	+/+
Extension contact (Extfreq)	Number of contacts	+/+
Livestock possession (TLU)	Number (Total livestock units)	+/+
Farmer field day participation (FAFDP)	Dummy (1 if participated, 0 otherwise)	+/+
Training attendance (FAT)	Dummy (1 if trained, 0 otherwise)	+/+
Demonstration trials participation (PDT)	Dummy (1 if participated, 0 otherwise)	+/+

#### 3.6.1 Independent Variables

##### 3.6.1.1 Gender

Gender is measured as a dummy variable, where 1 indicates a male‐headed household and 0 otherwise. In smallholder farming households, both men and women actively participate in *teff* production. Due to prevailing sociocultural norms, men often have greater mobility and are more likely to attend community meetings, training sessions, and extension events, giving them better access to agricultural information. This can lead to higher adoption rates among male‐headed households (Wake 2018). (Asule et al. 2024), strengthening awareness creation and gender‐sensitive dissemination pathways is critical to achieving inclusive adoption outcomes.

##### 3.6.1.2 Family Size (Famsz)

Family size is treated as a discrete variable and measured in terms of adult equivalents—individuals capable of participating in agricultural activities. A larger family size can provide the necessary labor at critical times, helping ensure the timely execution of tasks according to the crop calendar. Recent studies have highlighted the importance of household labor availability in adopting and performing labor‐demanding crops like *teff* (Assefa et al. 2025).

##### 3.6.1.3 Distance to Seed Source (Dist)

This is a continuous variable measured in terms of walking hours from the household to the seed sources. Input suppliers such as seed dealers and agrochemical vendors are often concentrated near seed sources, making it easier for nearby farmers to access necessary agricultural inputs. Distance from the nearest market has been shown to negatively affect technology adoption (Feyissa et al. 2025).

##### 3.6.1.4 Year of Schooling (EDU)

Education level is recorded as a dummy variable, taking the value of 1 if the household head is literate and 0 otherwise. Literate farmers are more likely to understand and process new information, access extension services, and respond to market signals. This enhanced capacity allows them to make informed decisions regarding the adoption of improved practices and technologies. As a result, education contributes to increased willingness to adopt innovations, which can ultimately lead to higher *teff* productivity (Asante et al. 2024).

##### 3.6.1.5 Age

Age is treated as a continuous variable, measured in years. Age may be associated with a lower likelihood of adopting new technologies, as older farmers might be more risk‐averse or less open to change. Several studies have found that age tends to have a negative influence on the adoption of agricultural innovations (Habane and Duale 2025), suggesting that younger farmers may be more responsive to new ideas and practices, including improved *teff* technologies.

##### 3.6.1.6 Land Holding (LH)

This is a continuous variable measured in hectares. It is hypothesized that there is a positive relationship between landholding size and the adoption of improved *teff* seed. Larger landholdings may enable farmers to allocate a portion of their land to try new technologies without jeopardizing their entire production. (Teshome and Tegegne 2020) found that the size of cultivated land significantly influences the likelihood of adopting improved agricultural technologies, including in cereal crop production.

##### 3.6.1.7 Farming Experience (Expre)

This is a continuous variable measured in terms of the number of years the household head has been engaged in *teff* farming. It is hypothesized that farmers with more years of farming experience are more likely to adopt improved *teff* technologies and to use them more intensively. Previous studies, such as (Tekeste et al. 2023), have shown a positive link between farming experience and technology adoption probability.

##### 3.6.1.8 Access to Credit (AC)

This variable is measured as a binary (dummy) variable, taking the value of 1 if the household has access to credit and 0 otherwise. Access to credit is expected to positively influence the adoption of improved *teff* technologies. When credit is accessible, households are less constrained by liquidity limitations and are more likely to invest in new and potentially high‐return technologies. Recent studies, such as (Workineh et al. 2020), have confirmed that access to credit significantly enhances the likelihood of adopting improved agricultural practices in Ethiopia.

##### 3.6.1.9 Frequency of Extension Contact (Extfreq)

This is a continuous variable measured by the number of times a respondent interacts with agricultural extension agents in a given year. Effective adoption and use of agricultural technologies rely heavily on the communication between extension agents—who serve as facilitators of innovation—and farmers at the grassroots level. Recent empirical evidence, such as (Alam et al. 2024), support the notion that regular extension contact positively influences both awareness and adoption of agricultural innovations among smallholder farmers in Ethiopia.

##### 3.6.1.10 Livestock Possession (TLU)

This variable is measured in Total Livestock Units (TLU), which standardizes different types of livestock into a common unit. Livestock ownership is hypothesized to have a positive relationship with the adoption of improved agricultural technologies, including those for *teff* production. Livestock often serves as a proxy indicator of a household's wealth and asset base, enabling farmers to manage financial risks associated with new technology adoption. (Tessema et al. 2018) confirm that livestock ownership significantly influences technology adoption among smallholder farmers in Ethiopia.

##### 3.6.1.11 Farmer's Field Days Participation (FAFDP)

This variable reflects whether a farmer has participated in agricultural field days focused on improved *teff* varieties. Field days serve as effective platforms for experiential learning, allowing farmers to observe the performance of improved varieties, interact with experts, and engage in peer‐to‐peer discussions and are expected to positively influence the adoption of demonstrated technology. (Hussain and Maharjan 2025) have shown that attendance at field demonstrations significantly improves the adoption of improved agricultural technologies by enhancing farmers' knowledge and perception of benefits.

##### 3.6.1.12 Farmer's Attendance to Training (FAT)

Farmer training is a critical component in providing farmers with access to new agricultural knowledge, which can influence their decisions to adopt improved technologies. These sessions provide farmers with practical skills for managing both inputs and outputs more efficiently. More recent studies, such as (Mgendi et al. 2022), have further emphasized that farmer training plays a significant role in improving adoption rates by broadening knowledge, fostering confidence, and providing necessary skills for managing new agricultural technologies.

##### 3.6.1.13 Participation in Demonstration Trials (PDT)

Farmer participation in demonstration trials is expected to increase the likelihood of adopting new technologies, as it allows farmers to directly observe the benefits of the demonstrated practices. By participating in these trials, farmers can better assess the viability and productivity of the technology, which often leads to increased adoption. Demonstration trials are effective tools for promoting the uptake of agricultural innovations, especially in smallholder farming systems (Mgendi et al. 2022).

## 4 Results and Discussion

### 4.1 Descriptive Statistics of Continuous Variables by Adoption Status

The descriptive statistics of the variables of the sample household investigated are illustrated in Table 2. The independent two‐sample *t*‐test mean comparison of the continuous variable with the adoption decision of the sample households indicates that farm experience, farm size, family size, extension contact, livestock owned, and on‐farm income have statistical mean differences across the adopter and nonadopter households.

**TABLE 2:** Mean of farm and farmers' characteristics of adopters and nonadopters.

Variables	Adopters (*Y* = 1)			Nonadopters (*Y* = 0)			*t*‐test
	Obs	Mean	Std. dev.	Obs	Mean	Std. dev.	
Age	276	45.300	7.910	108	44.704	9.637	−0.62
Farm experience	276	15.010	5.554	108	9.607	7.988	−7.49***
Farm size	276	0.798	0.601	108	0.463	0.230	−5.64***
Family size	276	5.430	1.394	108	4.759	1.611	−4.12***
Extension frequency	276	16.896	4.582	108	11.027	4.897	−10.9***
Distance	276	3.609	1.241	108	3.662	1.139	0.44
Livestock owned	276	4.669	1.742	108	6.171	10.175	2.40**
On farm income	276	41,766	19,828	108	30043.9	15,119	−5.47***

*Note:* *** and ** indicate significance at 1% and 5% levels, respectively.

The dataset in Table 3 includes 384 farm households of which approximately 71.88% were adopters, meaning they planted at least one of the improved *teff* seeds on their farmland.

**TABLE 3:** Improved *teff* seed adoption status of farmers.

Adoption‐status	Freq	Percent
Nonadopters	108	28.13
Adopters	276	71.88

### 4.2 Descriptive Statistics of Dummy Variables According to Adoption Status

Table 4 illustrates the proportional test of the categorical variable with the adoption status of sample households. The proportion test revealed that gender, participation in demonstration, farmer field day, access to credit, and credit access have a significant associations among adopter and nonadopter respondents. The results revealed that the major sample respondents (96.7%) were male‐headed households, while 3.3% were female‐headed households from adopter categories. The proportions of male‐headed households for adopters and nonadopters categories were 96.7% and 85.2%, respectively. The variable is statistically and significantly (*p* < 0.01) associated with the adoption status of the sampled households. Data are often collected on whether a farmer had access to field day participation, demonstration participation, or training.

**TABLE 4:** Description of dummy variables according to farmer adoption status.

	Adaptors (*Y* = 1)	Nonadopters (*Y* = 0)	Chi‐2 *p*‐value
Sex			
Female	9	16	0.000
Male	267	92	
Farmer training			
Yes	215	84	0.980
No	61	24	
Demonstration participation			
Yes	184	88	0.004
No	92	20	
Field day participation			
Yes	154	33	0.014
No	122	75	
Education level			
Literate	222	45	0.000
Illiterate	54	63	
Credit access			
Yes	211	70	0.021
No	65	38	

Regarding institutional characteristics, around 55.98% of adopters had access to field day participation to cultivate improved *teff* varieties. However, the provision of training had no significant association between adopters and nonadopters. For demonstration participation, around 48% of adopters had access to it, whereas 6.25% of nonadopters did not. The study found highly significant (*p* < 0.05) differences between adopter and nonadopter households in access to field day participation and demonstration participation, indicating that those with access to these resources are more likely to adopt the technology.

Education is one of the explanatory variables in this study, as it aids in accessing, understanding, processing, and applying various agricultural information. Education is categorized as literate and illiterate. The Chi‐square test confirms the existence of a significant difference in the level of education between adopters and nonadopters of improved *teff* seeds.

Credit availability was found to be as an essential economic determinant in the purchasing agricultural inputs, particularly for farmers who lack sufficient funds. Amhara Credit and Savings Institution (now a day Tsedey Bank) has been assisting farmers by offering various forms of credit for agricultural inputs. Furthermore, a farmers' cooperative was established, which serves as a key resource for farmers by providing agricultural inputs on a credit basis, acting as a key resource for farmers. As a result, the availability of loans has significantly encouraged farmers to adopt new technology. As shown in Table 4, a higher percentage of respondents (76.4%) use credit for improved *teff* production in the adopter category, and the difference between adopter and nonadopter households is statistically significant (*p* < 0.05).

**FIGURE 3:**
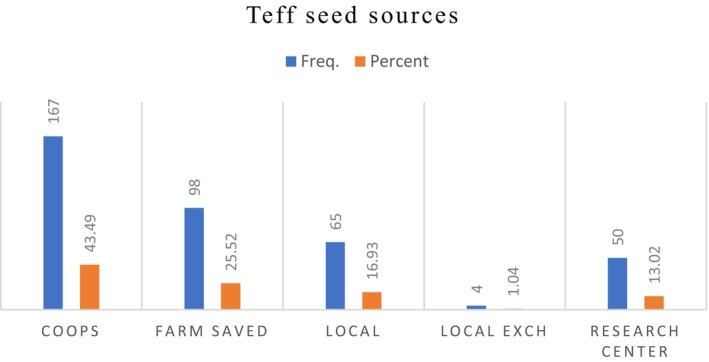
Sources of improved *teff* seed varieties. *Source:* Own survey, 2024.

Regarding Sources of improved *teff* varieties, a significant number of farmers, approximately 43%, reported that they obtained improved *teff* seeds from local cooperatives supplied by Amhara Seed Enterprise, while 25.52% relied on their own seeds from previous harvests as a source of improved *teff* varieties (Figure 3).

#### 4.2.1 Sources of Extension Services

**FIGURE 4:**
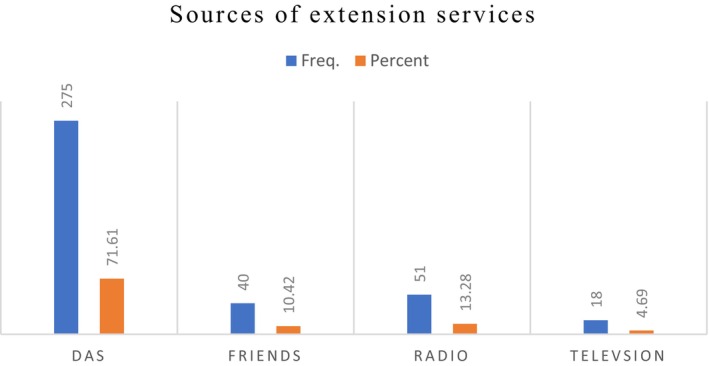
Source of extension services. *Source:* Own survey, 2024.

The survey result indicates that, of the total respondents, 71.61% obtain extension services from development agents (DAs), while 13.28% of the respondents obtain the service from radios, which is the second‐most common source of extension services (Figure 4).

**FIGURE 5:**
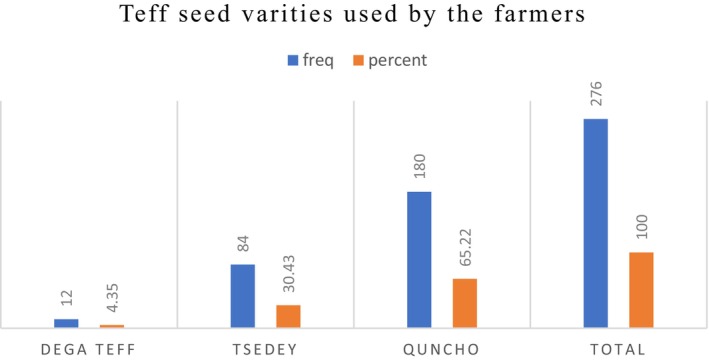
Varieties of *seed* used by the farmers. *Source:* Survey result 2024.

*Teff* seed varieties used by the farmer's sampled household heads in the study area used different types of *teff* seed for production. The result in Figure 5 indicates that the majority of households, 180 (65.22%), cultivate an improved *teff* seed variety called Quncho, while 84 (30.43%) cultivate Tseday and 12 (4.35%) cultivate Dega, respectively, from the adopter category.

**FIGURE 6:**
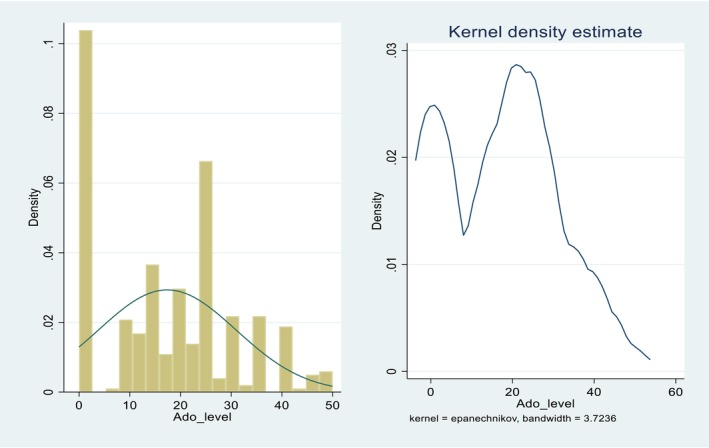
Distribution of improved *teff* adoption intensity.

Figure 6 below shows both the histogram distribution and kernel density estimates of improved *teff* seed used in kg per hectare. The analysis revealed, on average, farmers who adopted improved *teff* seed applied 27.97 kg per hectare with a standard deviation of 11.99 kg/ha, indicating a wider variation in usage among this group. These figures suggest that adopters not only use significantly more improved seed but also vary more in how much they apply, possibly reflecting differences in access, farm size, or individual farming strategies.

The adoption intensity of improved *teff* seeds, as illustrated from Figure 6, larger proportion of sampled respondents adopt improved *teff* seeds at a low level concentrated around zero. This indicates that many households either do not adopt improved *teff* varieties or do so at very minimal levels. A secondary concentration observed around 25 marks implies that a small group of respondents adopt at a moderate level. Very few sampled respondents show a high level of adoption beyond 40 on the adoption intensity of improved *teff* seeds.

Table 5 presents the average kg of *teff* seed adopted (only adopters) given that sample households have already adopted and compares the value with the predicted (Adoption predicted) and the true value of the dependent variable (adoption intensity) for all observations. As expected, we found that the sample average prediction (21.85) is higher for those sample households who have already adopted improved *teff* seeds.

**TABLE 5:** The comparison of the true, predicted, and average value of the adopters.

Variable	Obs	Mean	Std. dev.	Min	Max
Adoption Intensity	384	17.346	13.619	0	50
Adoption predicted	384	17.382	10.167	0.025	40.882
Only adopters	384	21.846	6.121	4.744	41.363

### 4.3 Econometric Analysis

#### 4.3.1 Determinants of Adoption Decision of Improved *Teff* Seed

Table 6 presents the effect of demographic, institutional, and socioeconomic factors affecting adoption decisions and intensity of use of improved *teff* seed.

**TABLE 6:** Econometric analysis of determinants of adoption and extent of adoption of improved *teff* seeds.

Variables	1st hurdle (Decision)		2nd hurdle (intensity)	
	dy/dx	Std. err.	Coefficient	Std. err.
Gender	−0.004	2.353	−1.013	2.981
Farming experience	0.280***	0.086	−0.029	0.109
Credit access	−0.256	1.080	0.459	1.255
Age	−0.295***	0.071	−0.142*	0.086
Education level	7.920***	1.065	6.804***	1.396
Farm size	8.399***	1.164	8.603***	1.144
Participation in a demonstration	2.173**	1.074	2.039*	1.180
Participation in the farmer's field day	2.420**	1.035	1.976*	1.169
Participation in farmer training	−0.317	1.179	−0.149	1.349
Family size	0.629*	0.345	0.036	0.391
Extension frequency	0.611***	0.098	0.280**	0.122
Distance	0.067	0.435	0.181	0.482
Income (log)	7.400***	2.389	4.563*	2.722
Total livestock unit (TLU)	—	—	−0.187	0.328
_cons	−8.731	2.423	−7.822	13.174
Lnsigma				
_cons	2.130***	0.045		
/sigma	8.422	0.386		

*Note:* ***, ** and * indicate significance at 1%, 5% and 10% levels, respectively.

The marginal effect of farm experience is 0.014 percentage point and is statistically significant at the 10% level, indicating that each additional year of farming experience increases the likelihood of adopting improved *teff* seed by approximately 1.4 percentage points, holding other factors constant. This suggests that more experienced farmers are slightly more likely to adopt improved seeds, likely due to their accumulated knowledge, better risk assessment, and greater exposure to agricultural innovations over time. Although the effect is modest and only marginally significant, it still highlights the role of experience in influencing adoption decisions among farming households. The result is in harmony with the recent study by (Tekeste et al. 2023), which provides empirical evidence that farming experience was a significant determinant of the adoption of improved varieties of wheat, *teff*, and maize in central Ethiopia.

Age of the sampled household head has a statistically significant and negative effect on the likelihood of adoption of improved *teff* seeds, with a marginal effect of 0.295 percentage points and a *p*‐value of less than 1% level. This implied that for a 1‐year increase in the age of the household, the probability of adopting improved *teff* seeds decreased by 29.5% at ceteris paribus. As compared to the younger farmer, older farmers are reluctant to accept new technology, risk‐averse, more reliant on traditional farming habits, and refuse to invest in long‐term productivity‐enhancing technologies. The result is consistent with the recent empirical findings by (Habane and Duale 2025; Endalew et al. 2024; Tekeste et al. 2023), which demonstrated the age of the household as a key determinant of the adoption of improved agricultural technology.

The model result revealed that the level of education in a literate household, compared to an illiterate household, has a positive and significant effect on the adoption of improved *teff* seed at less than 1% level, with a 7.92 percentage points indicating a strong and reliable relationship. This strong positive effect is justified by literate farmers can interpret the extension message more clearly, have access to comprehensive agricultural information (weather conditions, price signal), follow extension‐recommended agricultural practices, and have communication with service providers. The result aligns with (Endalew et al. 2024; Tekeste et al. 2023).

The regression result indicates that farm size has a positive and statistically significant effect on the likelihood of adoption of improved *teff* seeds, with a *p*‐value at less than 1% level, a marginal effect coefficient of 8.36 percentage points. It implied that farm size was a key determining factor of adoption of improved *teff* seed, together with other factors like education level, participation in demonstration, field day, and extension frequency. The positive effect is thought to arise because farmers with large farm sizes can cope with risks and the initial cost of adopting newly released technologies.

Participation in demonstrations has shown a statistically significant effect on the likelihood of adopting improved agricultural technologies. Farmers who participated in demonstration plots were found to positively influence the adoption of improved *teff* seed varieties, with the marginal effect of 2.173 percentage points and a *p*‐value less than 5%, indicating that farmers exposed to hands‐on demonstration are more likely to gain first‐hand information about the benefits of these technologies. Likewise, participation in farmer‐field day had a significant and positive effect of 2.42 percentage points (*p* < 0.05), implying that households that attend farmer‐field‐day sessions are more likely to adopt improved *teff* varieties than nonparticipant farmers. This finding underscores the benefit of participatory outreach in raising awareness, building trust, and hands‐on familiarity with improved agricultural technologies. The finding is consistent with the study by (Hussain and Maharjan 2025), suggesting that farm demonstration enhances agricultural yield, technology adoption, and farm profitability in Pakistan. (Mgendi et al. 2021) also highlighted their role among Tanzanian rice farmers. In addition, the finding on farmer field day participation is consistent with the study by (Emerick and Dar 2021). Socioeconomic and institutional determinants were also found to significantly influence adoption decisions in similar studies, such as (Musafiri et al. 2022a) in Western Kenya. In addition, as (Njenga et al. 2021) emphasize, participation in demonstrations and access to communication channels significantly boost farmers' decision to adopt new technologies.

The study confirms that family size measured in adult equivalent has a statistically significant and positive effect on the adoption of improved *teff* seeds at a *p*‐value of less than 10% level with a marginal effect of 0.629 percentage points. It implies that each additional family size unit significantly increases the likelihood of the adoption of improved *teff* seed in the study area. The possible explanation is that the nature of the crop is labor‐intensive during the period of planting, weeding, and harvesting, which demands a substantial number of laborers to manage it.

Furthermore, extension contact measured by the number of sampled households interacted with the development agents (DAs) was the main driver of improved *teff* seed adoption decisions. The model results revealed that the marginal effect for extension contact is 0.611 percentage points with a *p*‐value less than the 1% level, which confirms a highly significant relationship between extension contact and adoption decisions. Agricultural extension services increase technology adoption by 4.2% (Alam et al. 2024). Similar to the findings by Musafiri et al. 2022a, 2022b, agronomic interventions like improved seed adoption are more successful when supported by accessible inputs and extension support.

The research finding affirms that the household income measure in the logarithm of annual farm income has a positive and significant effect on the probabilities of adopting improved technologies. The marginal effect of household income was 7.4 percentage points with a *p*‐value less than 1% level, which plays a significant role in increasing farmer capacity to purchase and adopt improved agricultural technologies, including *teff* seeds. The marginal coefficient (*β* = 7.4, *p* = 0.000) means that a 1% increase in income results in a considerable rise in the likelihood of adoption of improved *teff* seeds, hence it increases farmers' economic capacity to access costly inputs like certified seed and fertilizers. The result is aligned with the finding by (Nonvide 2020).

#### 4.3.2 Factors Affecting the Intensity of Use of Improved *Teff* Seed

The effect of the age of the household head on the extent of adoption was negative and statistically significant (*p* < 0.05 level). Aged people may no longer find it useful to allocate more land for improved seeds, as they may feel that their future is behind them, leading to a lack of motivation or a preference to be satisfied with existing improved seeds. Furthermore, producers often allocate a plot of land to their children when they are young enough, decreasing the availability of land for adopting newly released agricultural technology. As a result, young individuals are more inclined to support and use enhanced *teff* seeds more intensively. Research by (Teshome and Tegegne 2020) demonstrates that, at a 5% probability level, age had a negative and significant impact on the intensity of use of improved *teff* types.

The level of education plays a crucial role in changing the mindset on the optimal level of adoption of improved agricultural technology. The literacy variable had a positive coefficient, indicating that literacy had a beneficial impact on the intensity of adoption of improved *teff* seeds at *p* < 0.01 with a coefficient of 6.84. This study revealed that literate farmers were the most likely to adopt improved *teff* seeds, while other fixed variables remained constant. Furthermore, having a formal or informal education enhanced the likelihood of having access to, and thus adoption of, improved *teff* seeds. This result is consistent with the previous work by Mahoussi et al. (2021).

The study further reveals that the coefficient of the farm size variable is significant at *p* < 0.01 and positively correlated with the adoption of improved *teff* seeds with a coefficient of 8.60. The farmer with a large land holding increases farmer initiative to experiment with new technology on their land before adopting and ultimately increases the intensity of adopting improved *teff* seed on a large scale. These findings align with (Teshome and Tegegne 2020). The proportion of cultivated land also found a positive and significant influence on the probability of adoption of high‐yielding *teff* seed at a less than 1% significant level (Milkias 2020). In addition, the finding is consistent with (Mwaura et al. 2022), farm size was positively associated with adoption intensity.

Participation in demonstration was the most important independent variable determining the extent of adoption of improved teff seed at the smallholder level. The regression analysis was positive and statistically significant at less than a 10% significance level, with a coefficient of 2.04, implying that farmers who participate in the demonstration site are more likely to adopt them more intensively. It serves as an experimental learning platform and exposes farmers to the practical application of newly released *teff* varieties like Quncho, Tsedey, and Dega *teff*, and farmers can have first‐hand information. According to (Mgendi et al. 2022; Singh et al. 2018), on‐farm training and demonstration create awareness, knowledge, peer‐to‐peer learning, and confidence to adopt agricultural technologies.

Furthermore, the regression result demonstrates that participation in field days significantly and positively influences the intensity of adoption of improved *teff* seeds, with a coefficient of 1.98, *p* < 0.1. The farmers who participate in strategically organized agricultural occasions are more likely to adopt more intense seed varieties than nonparticipants. In field day participation, not only do agricultural experts present technical aspects, but there is an information and knowledge sharing among fellow farmers who have tried the technology before. Field day participation also addresses information asymmetry and enhances interactive learning, which benefits farmers with limited literacy and weak extension infrastructure that inhibit information flow. The finding is in line with (Emerick and Dar 2021).

In addition, the frequency of extension contact has a positive and statistically significant effect on the intensity of adoption of *teff* seeds; the coefficient is 0.28 with a *p*‐value of less than 5% level, indicating a significant influence at less than 5% level. It implies that farmers who have frequent extension contact with development agents (DAs) are more likely to adopt *teff* seeds intensively. Agricultural extension services delivered by extension agents provide technical support, practical advice, and dissemination of new knowledge to farmers. The result is consistent with (Yitayew et al. 2021), which confirms that extension contact increases the likelihood and intensity of use of improved technologies.

The model result illustrated that the logarithmic income of farmers has a positive and statistically significant marginal effect on adoption intensity of improved *teff* seed at the 10% level, with a coefficient of 4.56. It means that a unit increase in farmer income in Birr is associated with a substantial increase in adoption intensity by 4.52 kg of *teff* seed. Higher income reduces financial liquidity constraints upon purchasing and adopting new technologies, including improved *teff* seed, fertilizer, and pesticides.

## 5 Conclusion and Recommendation

The study aimed to identify influencing factors determining adoption decisions and the intensity of adoption of improved *teff* seed among smallholder farmers in the Jabitehnan district of the Amhara region, Northwest Ethiopia. The choice to adopt and the intensive use of improved *teff* seed technology were affected by a combination of demographic, socioeconomic, and institutional factors. The descriptive results revealed that out of the total sample of households, 276 (71.87%) households are adopters of improved *teff* seed, while 108 (28.27%) were nonadopters.

The first hurdle estimation has shown that farming experience, education level, farm size, participation in demonstration plots, field day participation, family size, extension frequency, and household income have a positive and significant effect on the adoption decision of improved teff seed, while the age of the sampled household had an adverse effect on the probability of adoption of improved *teff* seeds. The second hurdle (intensity) estimate results imply that the intensity of use of improved *teff* seed is positively and significantly affected by education level, farm size, participation in demonstration plots, farmer field days, frequency of extension contact, and farmer income, while the age of the household had a negative and significant relationship with the intensity of adoption of improved *teff* seed.

According to the findings of the study, the following suggestions have been forwarded for policymakers and agricultural extension systems to take initiative in promoting the use of improved *teff* seed technology.

First, farm experience plays a central role in the adoption of improved teff seed by producing practical‐oriented farmers, which increases their rationality in using improved agricultural technology and positions farmers to implement practices effectively. Therefore, policymakers and agricultural agents should prioritize capacity‐building training that leverages farmers' experience.

Second, as the age of the sample households negatively influences the adoption decision and the intensity of adoption, age‐sensitive extension programs should be designed, developing mechanisms such as group‐based demonstrations and farmer‐to‐farmer mentoring systems—pairing older farmers with younger ones—to facilitate peer learning and encourage the adoption of labor‐saving technologies, especially among older farmers.

Third, efforts should be made to increase and institutionalize the intensity of adoption of improved *teff* seed and make it easier for farmers to access government programs and services. Raising farmers' knowledge levels through rural adult literacy programs integrated with agricultural training, demonstrations, and field days can help farmers understand basic *teff* production techniques.

Fourth, to enhance agricultural productivity, the pooled‐land approach, similar to cluster farming practices, should be strengthened and implemented. Larger, consolidated farming units facilitate the provision of extension services, training, disease management, and effective monitoring of agricultural production.

Fifth, empirical findings suggest that policymakers and development agents should expand farmer demonstration projects and participation in field days across different ages, genders, and farm sizes to build trust in improved *teff* seeds and promote widespread adoption.

Sixth, to address labor constraints faced by small families during critical farming operations, policymakers should promote labor‐sharing cooperatives and community‐based workgroups like Wonfel and Debo (traditional labor cooperation), which can increase farmers' willingness to adopt improved *teff* seeds.

Seventh, to boost adoption and intensive use of improved *teff* technology, policymakers should emphasize location‐specific, crop‐specific, and weather‐sensitive extension services using various platforms such as mobile phones, radio, newspapers, magazines, and television. Strengthening ongoing farmer‐agent interactions is crucial to scaling up the use of improved *teff* seed, ultimately improving productivity and livelihoods among smallholder farmers in the study districts.

Finally, the findings highlight the importance of income in the adoption and use of improved *teff* seeds. To enhance the use and intensity of its use, policymakers should develop income‐generating strategies for rural households, such as supporting off‐farm activities, providing input subsidies, or targeted credits—measures that reduce economic barriers to adoption. Improving household income can improve livelihoods and create an environment conducive to sustained, intensive use of agricultural technologies in districts like Jabitehnan.

## 6 Limitations of the Study

First, because the data rely on self‐reported information from sample households about inputs used, participation in demonstrations, field days, training, use of improved teff seed, and extension activities, respondents may unintentionally provide inaccurate details or misremember, affecting the data's reliability. Additionally, participants might overstate their involvement in recommended agricultural activities, including extension participation, adoption of improved agricultural technology, field days, demonstrations, and training, to align with what they perceive as socially acceptable or expected by researchers or authorities. The second limitation of this research is that it relies entirely on cross‐sectional data, which collects and analyzes information at a single point in time. The third limitation is that, although identifying associations between demographic, socioeconomic, and institutional factors and farmers' adoption status is valuable, it does not allow for causal inference between these factors and the adoption or extent of using improved teff seeds. The fourth limitation is that this type of data does not account for dynamic environmental factors such as policy changes, market volatility, or weather patterns over time that could influence farmers' adoption behaviors. The last limitation is that this research is limited to Jabitehnan district in Northwest Ethiopia, focusing on area‐specific analysis. However, the results may not be applicable to other regions with different agroecology, cultures, or institutional contexts. Therefore, caution should be exercised when applying these findings to broader populations or regions without further validation.

## Author Contributions

**Gizachew Wosene:** conceptualization (equal), data curation (equal), formal analysis (equal), methodology (equal), software (equal), writing – original draft (equal), writing – review and editing (equal). **Yuhan Pang:** methodology (supporting), writing – review and editing (supporting). **Jianmin Cao:** conceptualization (equal), investigation (supporting), methodology (supporting), supervision (equal), writing – review and editing (supporting). **Arshad Ullah Jadoon:** methodology (supporting), writing – review and editing (supporting).

## Conflicts of Interest

The authors declare no conflicts of interest.

## Data Availability

The data is available from the corresponding author upon reasonable request.
